# A droplet-based microfluidic approach to isolating functional bacteria from gut microbiota

**DOI:** 10.3389/fcimb.2022.920986

**Published:** 2022-08-18

**Authors:** Jianan Yin, Xiuzhao Chen, Xiaobo Li, Guangbo Kang, Ping Wang, Yanqing Song, Umer Zeeshan Ijaz, Huabing Yin, He Huang

**Affiliations:** ^1^ Key Laboratory of Systems Bioengineering (Ministry of Education), Frontiers Science Center for Synthetic Biology, School of Chemical Engineering and Technology, Tianjin University, Tianjin, China; ^2^ New Technology R & D Department, Tianjin Modern Innovative TCM Technology Co. Ltd., Tianjin, China; ^3^ Institute of Shaoxing, Tianjin University, Zhejiang, China; ^4^ James Watt School of Engineering, University of Glasgow, Glasgow, United Kingdom

**Keywords:** gut microbiota, microfluidics, droplet, anaerobic culture, probiotics

## Abstract

Metabolic interactions within gut microbiota play a vital role in human health and disease. Targeting metabolically interacting bacteria could provide effective treatments; however, obtaining functional bacteria remains a significant challenge due to the complexity of gut microbiota. Here, we developed a facile droplet-based approach to isolate and enrich functional gut bacteria that could utilize metabolites from an engineered butyrate-producing bacteria (EBPB) of anti-obesity potential. This involves the high throughput formation of single-bacteria droplets, followed by culturing “droplets” on agar plates to form discrete single-cell colonies. This approach eliminates the need for sophisticated s instruments to sort droplets and thus allows the operation hosted in a traditional anaerobic chamber. In comparison to the traditional culture, the droplet-based approach obtained a community of substantially higher diversity and evenness. Using the conditioned plates containing metabolites from the EBPB supernatant, we obtained gut bacteria closely associated or interacting with the EBPB. These include anaerobic *Lactobacillus* and *Bifidobacterium*, which are often used as probiotics. The study illustrates the potential of our approach in the search for the associated bacteria within the gut microbiota and retrieving those yet-to-be cultured.

## Introduction

The gut microbiota plays a vital role in human health (Brody, 2020). Differences in the composition and function of gut microbiota are associated with a wide range of chronic diseases, from gastrointestinal inflammatory to metabolic, neurological, cardiovascular and respiratory diseases (Vijay and Valdes, 2021). With the rapid development of multi-omics technologies, such as metagenomics and amplicon sequencing, the complexity of the human gut microbiota and its role in disease are gradually being understood (Miyoshi et al., 2020). However, such methods are challenged by high cost, easily contaminated samples, and difficulties in reproducing experimental results (Kim et al., 2017). Moreover, its incapacity of obtaining and culturing isolates limits studying the interactions between species (Bilen et al., 2018). In contrast, the strains obtained by the culture methods can be used in *in vitro* and *in vivo* experiments, which are essential for the in-depth understanding of diseases and validation of potential therapeutics (Lagier et al., 2018). Therefore, in addition to culture-independent techniques, it is often necessary to isolate and obtain pure bacteria isolates for functional studies and verification.

Obesity refers to the condition in which excessive accumulation of body fat harms health, leading to shortened life expectancy and various problems (Hurt et al., 2010; Khan, 2016). Butyrate, a short-chain fatty acid, can decrease the pH of the intestine and promote the growth of beneficial bacteria in the gut (Chen and Walker, 2005). However, oral administration of butyrate compounds usually results in a low bioavailability (Bai et al., 2020). On the other hand, bacteria have been used as therapies for centuries, and recent advances in synthetic biology have unlocked tremendous opportunities for engineered bacteria in diagnosis and therapies (Riglar and Silver, 2018). Previously, we have engineered butyrate-producing bacteria (EBPB) and confirmed its potential anti-obesity effects on mice (Wang et al., 2022). Through metagenomic analysis of the gut microbiota, we found that after the long-term use of the EBPB bacteria, the abundance of beneficial bacteria, such as *Bifidobacterium*, *Lactobacillus*, and *Akkermansia*, increased. This indicates that the EBPB bacteria could regulate the microbiota composition, promoting the growth of beneficial gut bacteria. However, despite the knowledge of the genetic identity of these potential beneficial bacteria, obtaining these bacteria from gut microbiota *via* traditional culture has been futile and encountered well-known limitations, such as fast-growing bacteria dominating the culture plate. New approaches allowing enriching bacteria with different abundance and growth rates are highly desirable.

Droplet-based microfluidics has emerged as a powerful tool to control a small volume of fluid (e.g., pico- to nano-litre) in a high throughput manner and has found increasing applications in many fields (Sohrabi and Moraveji, 2020; Amirifar et al., 2022). Encapsulating individual cells in tiny droplets provides a protective environment for cells to grow without competition from others (Shang et al., 2017). In addition, thousands of single-cell microdroplets can be cultured in parallel, significantly enhancing the throughput (Mahler et al., 2021). It has been shown that droplet-based culturing enabled the growth of low-abundance bacteria (Watterson et al., 2020) and increased the diversity of cultured strains (Mahler et al., 2018). Furthermore, the surrounding oil can be pre-treated to tune the relative aerobic or anaerobic environment (Villa Max et al., 2020). Thus, the prospect of using droplet-microfluidic platforms to isolate and culture bacteria from the gut microbiota is very attractive. However, sorting desirable droplets requires bulk, sophisticated instruments, which are difficult to accommodate in a traditional anaerobic chamber.

Here, we developed a facile, droplet-based microfluidic approach to isolate and enrich individual bacteria cells from gut microbiota. This involves single-cell encapsulation in droplets followed by culturing the droplets on agar plates in an anaerobic chamber (which effectively “sorts” empty droplets). This approach can easily interface with conventional operations. Importantly, it can increase the diversity of obtained anaerobic species compared to traditional methods. Using desirable metabolites containing culture media, i.e. the supernatant from the engineered butyrate-producing bacteria (EBPB), we obtained metabolically functional species (e.g. *Lactobacillus* and *Bifidobacterium*), which could be used for further investigations, mining probiotics and constructing artificial flora to develop bacterial therapies.

## Materials and methods

### Strains and growth conditions

Engineered butyrate-producing BsS-RS06551 strain based on *Bacillus subtilis SCK6* host (EBPB) were created in-house previously (Wang et al., 2022). The butyrate yield reached 1.5 g/l, and the supernatant was weakly acidic. Green fluorescent protein (GFP) producing *Escherichia coli* BL21 was used to determine the most suitable cell loading density for single-cell droplet formation. *E. coli* BL21 and EBPB were routinely cultured in Luria-Bertani (LB) liquid medium and LB agar plates at 37°C. All strains obtained from faecal samples were cultured anaerobically in Yeast Casitone Fatty Acids (YCFA) liquid medium at 37°C and stored in a YCFA medium with 24% glycerol at -80°C. *Escherichia coli* Nissle 1917 (Biobw) and *Bifidobacterium pseudocatenulatum* (China General Microbiological Culture Collection Center, CGMCC) were routinely cultured in De Man, Rogosa and Sharpe (MRS) medium and MRS agar plates.

### Conditioned medium plates

A freshly transformed EBPB single colony on the LB agar plate was inoculated into 5 mL of liquid medium and cultivated at 37°C until the OD_600nm_ value reached 1.0. Then 2 ml of the bacterial broth was transferred into 200 mL of fresh LB medium and cultured at 37°C, 220 rpm for 24 h. The fermentation supernatants were collected by centrifugation of the culture at 12000 rpm for 10 min and filtering through 0.22 μm PES membrane to remove all bacterial cells. To form conditional medium plates (CMPs), an autoclaved YCFA medium with 3% agar was heated to 60°C, then mixed with the supernatant at a ratio of 1:1 and dispensed into disposable plastic plates. These plates were stored at 4°C after agar solidification.

### Fabrication of microfluidic chip

The microfluidic chip was designed using the AutoCAD 2016 software. A SU8 silicon mould was fabricated using the standard photolithography at the James Watt Nanofabrication Centre at the University of Glasgow, UK. Polydimethylsiloxane (PDMS) and curing agent (SYLGARD 184, Dow Corning Co., UK) mixture at a 10:1 ratio was poured onto the mould, degassed under a vacuum, and cured at 80°C for two hours. The PDMS replica was cut from the mould, and a biopsy punch (1.5 mm) was used to create both inlets and outlets for connecting tubes. After that, PDMS and a glass slide were ultrasonically cleaned with acetone, methanol, and isopropanol for five minutes and dried with filtered nitrogen gas. The channel side of the PDMS chip and the glass slide was treated in a Zepto plasma cleaner (Diener, Germany) for 20 seconds [p(O_2_):0.3~0.4 mbar] and immediately assembled. Finally, the chips were heated overnight in the oven at 80°C.

### Stool bacteria community preparation

The stool samples were collected from the previous animal experiments (Bai et al., 2020). Before each experiment, the anaerobic chamber (Shanghai Yuejin medical instruments Co. Ltd., HYQX-II) was filled with an anaerobic gas mixture (85% N_2_/10% CO_2_/5% H_2_) the day before. The stool samples stored at -80°C were taken out and transferred to the pre-set anaerobic chamber. After anaerobic treatment for two hours, the stool samples were dissolved and suspended in a pre-anaerobic treated YCFA medium. To prevent clogging of the microfluidic chip, the bacterial suspension was filtered through a 40 μm sieve to remove large food residues and particles.

### Bacteria encapsulation in droplets

The diluted bacterial suspension was used as the dispersed phase, and mineral oil (Sigma-Aldrich, light mineral oil) was used as the continuous phase. Various parameters (e.g., flow rates, cell loading density) have been evaluated to achieve robust droplet formation and single-cell encapsulation. The formed droplets were collected in an Eppendorf tube filled with mineral oil to prevent droplets from breaking. The microfluidic operation was conducted in the anaerobic chamber. In a typical experiment, a 0.03 g stool sample was dissolved in 4 ml of YCFA medium and filtered through a 40 µm membrane to remove large particles. Then the samples were washed three times, and the live cell density was measured using the Live/Dead BacLight Bacterial Viability kits (Invitrogen) and found to be 2.04 ± 0.06×10^7^/ml. Since the fluorescent dyes require oxygenation of the surrounding medium to fluoresce, we exposed the aliquot to air, which may affect the measured number of live cells.

### Inoculation of single-cell droplets and diluted cell solution

The number of samplings needed to characterize a microbiome completely can be addressed through Coupon Collector’s Problem (Morgan and Huttenhower, 2012). The approximate solution is given by Sampling cell number = N*(log(N) + 0.577216) + 1/2, where N is the total number of unique species in the microbiota. Recently the mouse gut microbial biobank revealed less than 150 species (Liu et al., 2020); thus, at least 414 cells are needed to characterize this level of diversity completely. To ensure sufficient sampling size while avoiding overseeding, the initial cell seeding number was chosen to be ~6.0×10^3^ cells per plate for all the conditions.

Thus, the concentration of droplets in the collection tube was measured using microscopy and adjusted to ~6.0×10^4^ droplets per µl with mineral oil. Since ~ 1% of droplets are single-cell droplets whilst the rest are empty droplets, 10 µl of droplets/oil solution was taken from the Eppendorf tube and spread on an agar plate for 72 h culture in an anaerobic chamber. Similarly, 10 µl of cell solution at 6.0×10^5^ live cells/ml was spread onto an agar plate in parallel and cultured for 72 hours in the anaerobic chamber. YCFA plates and YCFA plates containing the supernatant of EBPB were used, and five replicas per condition were conducted.

### 16S rRNA sequencing of gut microbiota

After 72 hours of culture, colonies cultivated at each plate were all scraped for 16S rRNA sequencing with primers targeting the V3-V4 regions to evaluate the diversity of the cultivated cells. The CTAB/SDS method was used to extract the total genome DNA in samples. DNA concentration and purity were monitored on 1% agarose gels. According to the concentration, DNA was diluted to 1 ng/µL with sterile water. 16S rRNA genes in distinct regions (16S V3-V4) were amplified with specific primer and barcodes. All PCR mixtures contained 15 µL of Phusion^®^ High-Fidelity PCR Master Mix (New England Biolabs), 0.2 µM of each primer and 10 ng target DNA, and cycling conditions consisted of a first denaturation step at 98°C for 1 min, followed by 30 cycles at 98°C (10s), 50°C(30s) and 72°C(30s) and a final 5 min extension at 72°C. Mix an equal volume of 1X loading buffer (contained SYB green) with PCR products and perform electrophoresis on 2% agarose gel for DNA detection. The PCR products were mixed in equal proportions, and then Qiagen Gel Extraction Kit (Qiagen, Germany) was used to purify the mixed PCR products. Following the manufacturer’s recommendations, sequencing libraries were generated with NEBNext^®^ Ultra™ IIDNA Library Prep Kit (Cat No. E7645). The library quality was evaluated on the Qubit@ 2.0 Fluorometer (Thermo Scientific) and Agilent Bioanalyzer 2100 system. Finally, the library was sequenced on an Illumina NovaSeq platform, and 250 bp paired-end reads were generated. The raw Illumina sequence data have been deposited in the NCBI database under BioProject accession number PRJNA861917.

### Bioinformatics & statistics

We have used VSEARCH to generate OTUs at 97% similarity using the protocol given in our previous study (Trego Anna et al., 2020), with one modification, we have used the latest version of SILVAMOD v138 reference database. Furthermore, as a prefiltering step, we removed typical contaminants such as those matching *Chloroplast* and *Mitochondria* and those that are unassigned (https://docs.qiime2.org/2022.2/tutorials/filtering/), which resulted in a final OTU table comprising of a total of 1,387 unique sequences for *n* = 17 samples. Statistical analyses were performed in R using the tables and data generated above, as well as the metadata associated with the study. We used the vegan package Field for alpha and beta diversity analysis (Oksanen et al., 2012). In particular, we used Rarefied Richness, a commonly used index, to measure the estimated number of OTUs within a sample after rarefying to the minimum library size. For beta diversity analysis, we have used Bray-Curtis distance in Principle Coordinate Analysis (PCoA) by using the cmdscale() function. Vegan’s adonis() function was used to perform an analysis of variance (PERMANOVA) of sources of variations (groups in this study) against Bray-Curtis distance as mentioned above. To find genera (OTUs collated at genus level) that are significantly different between multiple categories considered in this study, we have used DESeqDataSetFromMatrix() function from DESeq2 (Love et al., 2014) package with the adjusted p-value significance cut-off of 0.05 and log fold change cut-off of 2.0. This function uses negative binomial GLM fitting to obtain maximum likelihood estimates for the genera log fold change between the two conditions. Then Bayesian shrinkage is applied to obtain shrunken log fold changes, subsequently employing the Wald test for obtaining significances. For visualisation of results, we have used R’s package ggplot2 (Wickham, 2016).

### Genetic sequencing of selected isolates

To have a better resolution of species identification, 100 colonies formed at each condition were randomly picked for the full-length 16S rRNA gene sequencing (Sanger sequencing). Each picked colony was cultivated anaerobically in a 20 mL medium at 37°C for 12 hours. The genome of each single-cell colony was extracted using TIANamp Bacteria DNA Kit as a template for PCR. Universal primers 27F (5’-AGAGTTTGATCCTGGCTCAG-3’) and 1492R (5’-GGTTACCTTGTTACGACTT-3’) were used to amplify nearly the full length of the 16S rRNA sequence. PCR reactions were proceeded in 50 μL volumes, each containing 2 μL of 10 μM forward and reverse primers, respectively, 25 μL 2× Phanta Max Buffer, 17 μl ddH_2_O, 1 μl Phanta Max Super-Fidelity DNA Polymerase, 1 μl dNTP Mix and 2 μL DNA template. The thermocycling was performed as follows: 41 cycles (95°C, 15 s; 55°C, 15 s; 72°C, 60 s) after an initial denaturation at 95°C for 3 min, following a final extension at 72°C for 5 min. Then, the PCR products were purified and sequenced (Sanger sequencing) to get the gene sequences. Finally, the gene sequence was submitted to NCBI to identify the isolates. Sequence annotation and the database searches for sequence similarities were performed using the BLAST tool available online. Generally, these isolates’ 16S rRNA genes nucleotide sequences with homology between 99% and 100% with the reference strain in NCBI GenBank, belong to different strains of the same species (Janda and Abbott, 2007).

### Statistical analysis

All statistical analyses were performed using GraphPad Prism 8.3.0(538) (GraphPad Software, San Diego, California, USA, www.graphpad.com). Average data were given from five plates in each condition. For each condition, at least three independent repeated experiments were conducted.

## Results

### Production of stable and uniform droplets

**Figure 1:**
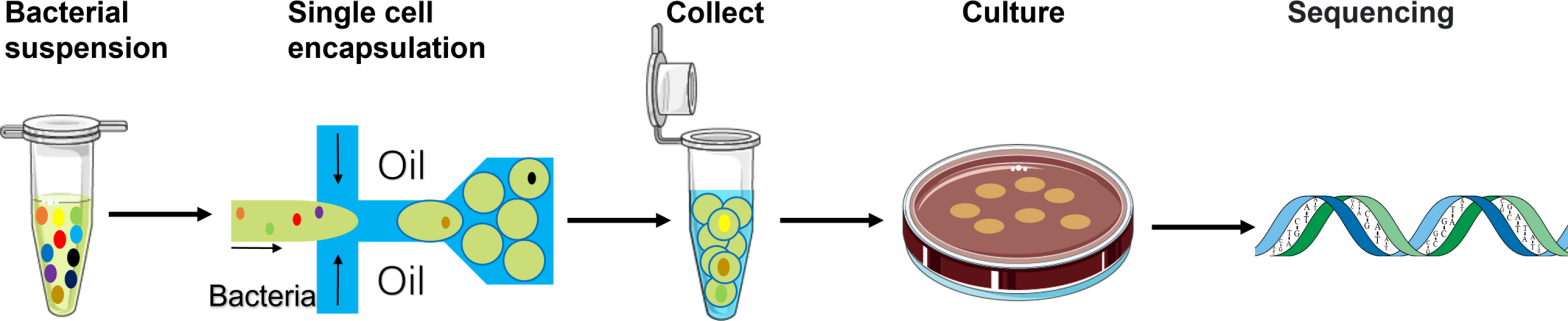
Schematic of the workflow using the single-cell droplet culture approach to search for functional bacteria from gut microbiota. Faecal samples were dissolved in media to extract gut microbiota into liquid. The pre-filtering bacterial suspension was injected into the microfluidic device for cell encapsulation in droplets. Stable and uniform droplets were collected into an Eppendorf tube containing mineral oil. Droplets were then spread on a culture plate and would burst eventually. The bacteria in the droplets could continue to grow on the plates. 16S rRNA sequencing technology was applied to identify growth colonies’ species.

The overall strategy is illustrated in **Figure 1**, and the setting for droplet formation is shown in **Figure S1**. A pre-filtered faecal bacterial suspension was injected into the microfluidic device as the aqueous phase to form water-in-oil droplets. A certain amount of the collected droplets were spread on an agar plate. In principle, every droplet containing a single cell could result in a single-cell derived colony if the cell is cultivable. Compared with the conventional series dilution-based culture, this method would offer simplicity and speed in obtaining single-cell derived colonies.

**Figure 2:**
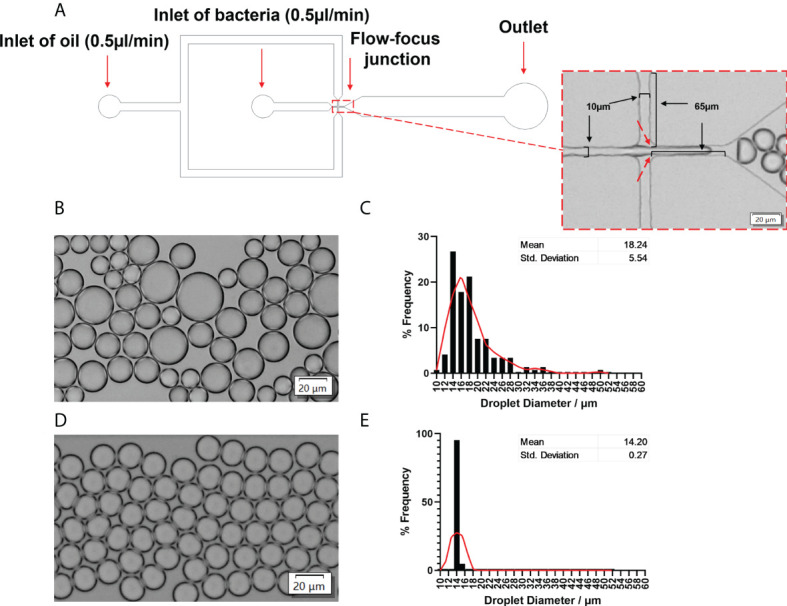
Microfluidic device to generate stable and uniform droplets. **(A)** Channel dimensions of the microfluidic chip. The dotted rectangle insert showed the bright-field image of the flow-focusing junction. A bacterial solution was the disperse phase, and oil was the continuous phase. Droplets were produced at the flow-focusing junction (indicated by red arrows). **(B)** Bright-field images and **(C)** the frequency distribution of droplet sizes produced by 2% Span80 in mineral oil. The average diameter was 18.24 ± 5.54 µm. **(D)** Bright-field images and **(E)** the frequency distribution of droplet sizes produced by 5% Span80 in mineral oil. The average diameter was 14.20 ± 0.26 µm. 94.2% of droplets diameter values fell into the bin of 14.05 µm. Randomly selected 150 droplets were measured using the cellSens imaging software. The relative frequency distribution (percentage) was analysed using GraphPad Prism 8.3.0(538). The red Lowess curve showed the trend of the data.

A flow-focused microfluidic chip was designed for droplet formation since it offers excellent flexibility to tune droplet size by varying the ratio between the continuous phase (oil) and the dispersed phase (aqueous) (Anna et al., 2003). Considering the size of bacteria cells (~ 1 μm), the cross-section of the chip had a dimension of 10μm (width) × 65μm (length) × 20μm (height) to facilitate small droplet formation and hence single-cell encapsulation (**Figure 2A**). We firstly evaluated conditions for the generation of stable and uniform droplets, which was essential for single-cell encapsulation. In this regard, the surfactant is an important factor since surfactants are adsorbed between the dispersed and continuous phases, thereby reducing the interfacial tension (Baret, 2012). This prevents droplets from coalescing with each other, therefore stabilizing the droplets in emulsion for a relatively long period (Mazutis et al., 2013). With 2% Span80 in the mineral oil phase, droplet coalescence occurred frequently; the droplet size had a wide distribution with an average value of 18.24 ± 5.54 μm (**Figures 2B, C**). Increasing Span80 concentration to 5% in mineral oil resulted in the generation of stable and uniform droplets at a throughput of 5500 droplets per second. Furthermore, hardly any coalescence was observed (**Figure 2D**), and more than 80% of droplets are 14.20 ± 0.27 µm in diameter (**Figure 2E**).

### Optimizing single-cell encapsulation

**Figure 3:**
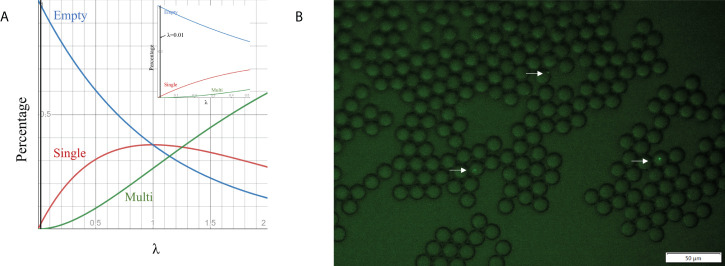
Cell encapsulation in droplets. **(A)** Relationship between the percentage of droplets containing different cell numbers and λ, which is the average number of cells per droplet volume. **(B)** Fluorescence images of droplet occupancy at 7×10^6^ cell numbers per ml (the most frequently used cell loading densities).

Cell loading density is an effective way to control the encapsulated cell number in droplets. Previous studies show that the number of cells encapsulated in droplets follows the Poisson distribution (Collins et al., 2015; Villa Max et al., 2020), which is given by *P*(*x*, *λ*) =*e^-λ^* (*λ^x^*/*x!*), where *x* is the number of cells per droplet, *P* is the proportion of droplets containing a given cell number *x*, and *λ* is the average number of cells per droplet volume (i.e. *λ*=*ρV*, where *V* is the droplet volume and *ρ* is cell loading density). We simulated *P* as a function of *λ* for empty droplets, single-cell droplets and multiple-cell droplets (**Figure 3A**). To ensure no more than one cell in each droplet (important for single-cell colony formation), we selected λ at 0.01, corresponding to ~ 1% of single-cell droplets and 99% of empty droplets. Based on the droplet dimension (14.20 ± 0.27 µm), the cell loading density was around ~7×10^6^ cells/mL. To validate the condition, GFP *E. coli* BL21 was used as the model strain to aid the detection of individual bacteria cells in a droplet *via* fluorescence imaging. No droplets with >1 cell were found (**Figure 3B**). The percentage of single-cell droplets is close to the theoretical value of 1%.

### Droplet-aided culture enhances the diversity of obtained species

The stability of the droplets spread on agar plates was monitored at 37°C over a time course (**Figure S2**). Most droplets remained intact for up to 8 hours (some were stable for up to 13 hours), indicating a single bacteria cell could grow first in droplets until the droplets broke. To test this, *E. coli* Nissle 1917 and *Bifidobacterium pseudocatenulatum* were cultured using the single-cell droplet culture and traditional dilution approach at the same seeding density for 48 hours. *Bifidobacterium pseudocatenulatum* is known to be strictly anaerobic and difficult to culture on agar plates. It was found that colonies of *B. pseudocatenulatum* only formed *via* the single-cell droplet culture (**Figure S3**). However, colonies of *E. coli Nissle 1917* formed under both conditions.

Furthermore, the colony formation rate of *E. coli via* the single-cell droplet culture is substantially higher than that of traditional culture. It is known that traditional plate cultures select fast-growing bacteria over slow-growing bacteria (i.e., many bacteria do not grow on commonly used culture plates) (Jannasch and Jones, 1959; Staley and Konopka, 1985; Olsen and Bakken, 1987). Similarly, in the case of faecal samples, substantially more colonies were formed *via* the single-cell droplet approach method (**Figure S4**). These results illustrated that our droplet approach can enhance cell growth, especially for slow-growing species, and challenge anaerobic gut bacteria.

**Figure 4:**
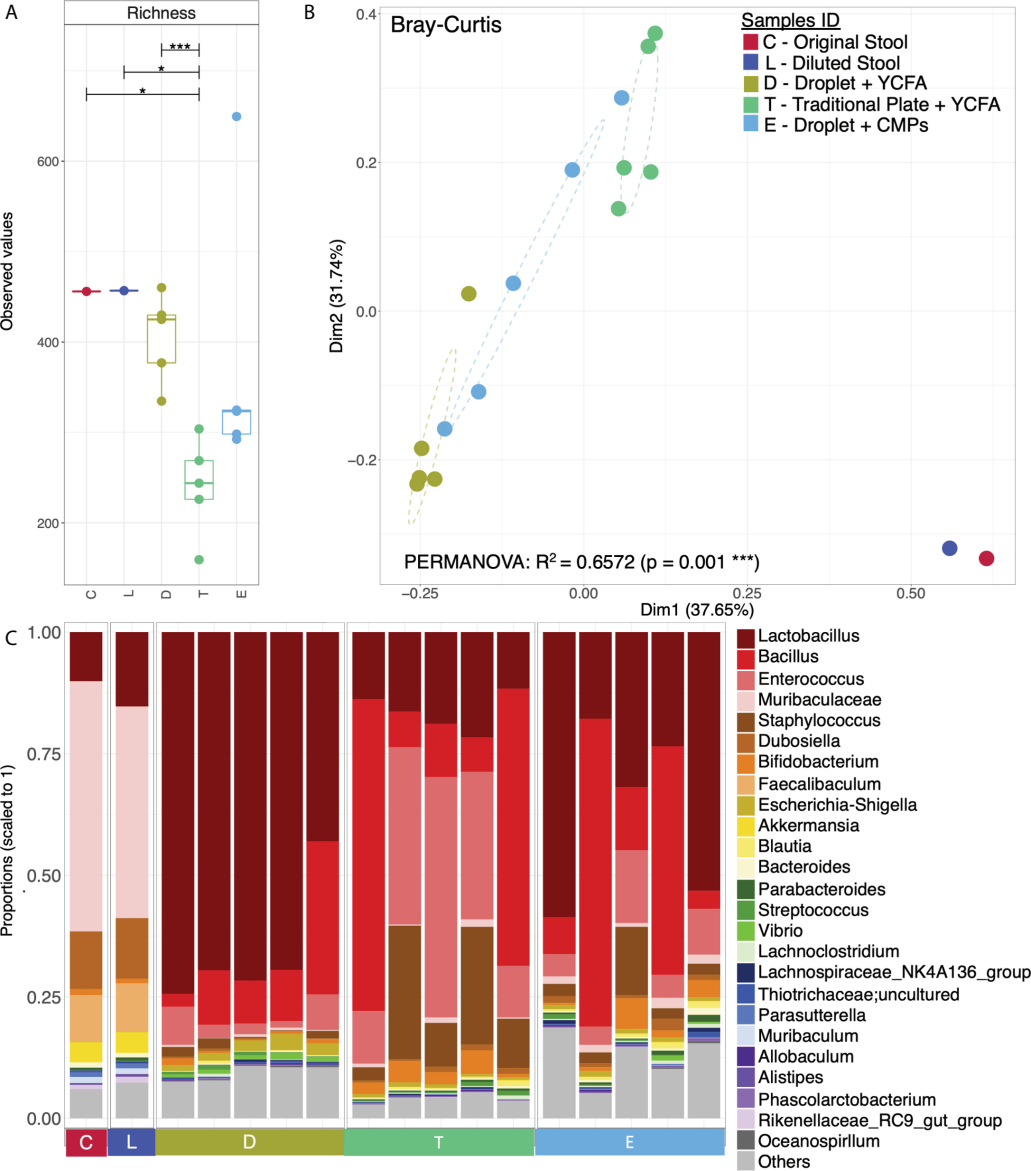
Microbial diversity and community structure. **(A)** Rarefied richness with lines connecting two categories where the differences were significant (ANOVA), i.e., * (p < 0.05), *** (p < 0.001); **(B)** Principle Coordinate Analysis (PCoA) using Bray-Curtis distance with the axis showing the percentage variability explained by each axis, and ellipses representing 95% confidence interval of standard error for each group (Sample IDs). PERMANOVA’s R^2^ represented percentage variability explained by the groups, i.e., 65.72%; and **(C)** Top 25 most abundant genera observed in all samples grouped by categories, where “Others” contain those genera which didn’t make the cut.

To understand the composition and diversity of the cultivated cells, all colonies under each condition were scraped for 16S rRNA sequencing with primers targeting the V3-V4 region. A total of 1,387 unique OTU sequences were found (n=17 independent experiments). Statistical analysis shows that the α − diversity (richness) of both the original faecal sample (denoted as **C**) and the diluted loading sample (denoted as **L**) was marginally significantly different from that of the cultured cells from the traditional culture (denoted as **T**) (P<0.05). However, the α−diversity of the cells from the droplet culture (denoted as **D**) is significantly different from that of the traditional culture **T** (p<0.001). Notably, there are no significant differences between either **C** and **D** or **L** and **D;** this suggests that the alpha diversity (richness) is conserved and makes us believe that the communities obtained from the droplet method are similar to the original communities we started with (**Figure 4A**).

**Figure 5:**
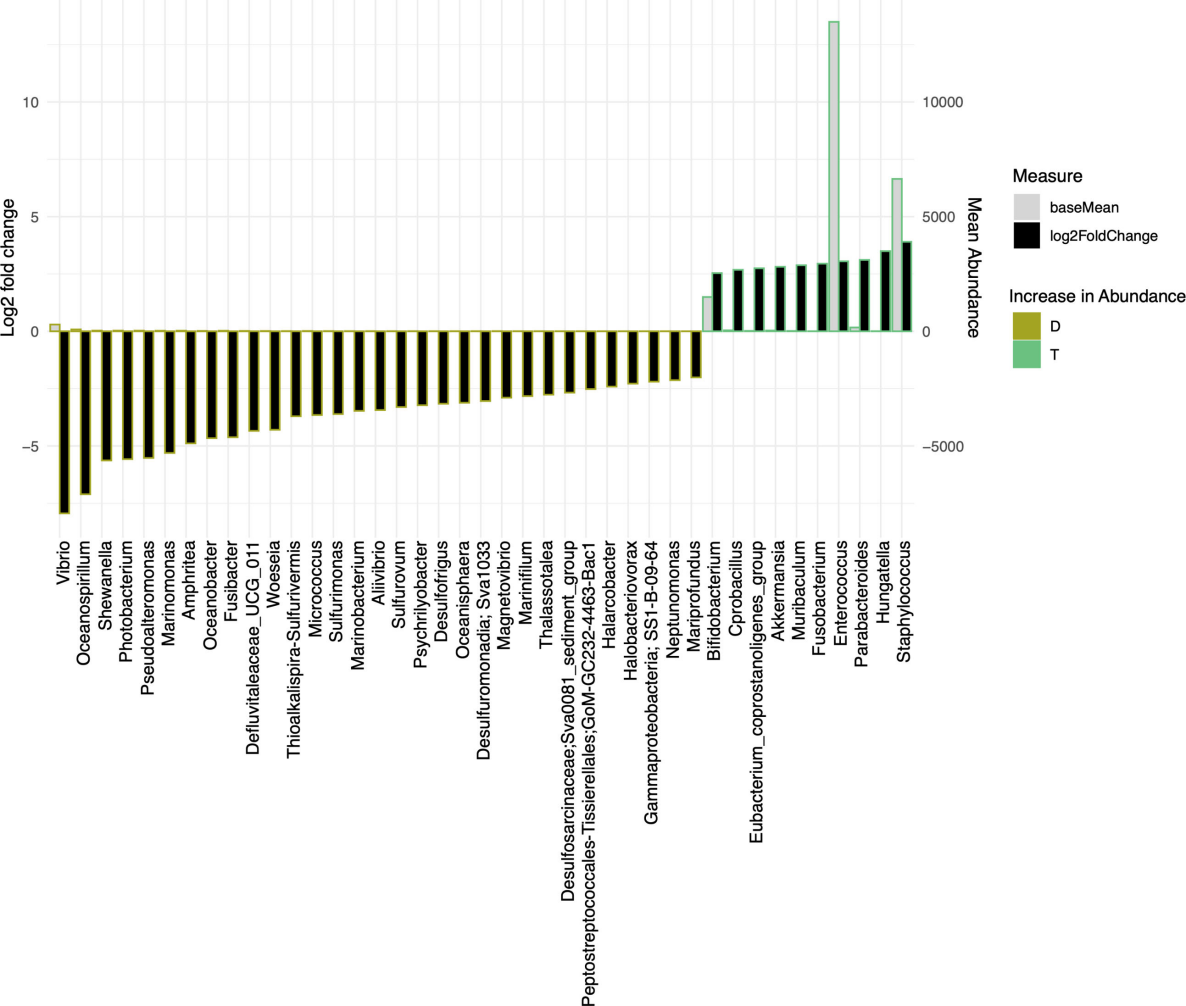
The bar charts show Log2 fold change in abundance of significant genera between groups (y-axis on the left and black bar) and the mean abundance across all the samples (y-axis on the right and light grey bar). Taxa increased in D (Droplet + YCFA) have bars with a mustard border (negative log2 fold change) meanwhile taxa increased in T (Traditional Plate + YCFA) have bars with a green border (positive log2 fold change).

We have also used Bray-Curtis distance for beta diversity analysis, which only considers the composition of community members. It was noticed that C and L were very close to each other, suggesting there was minimal loss of beta diversity between them. The samples from the traditional culture and those from the droplet method formed distinct clusters and did not overlap (**Figure 4B**), and PERMANOVA analysis showed 65% variability between all groups. The top 25 most abundant genera observed in all samples differed (**Figure 4C**). The differentially expressed genera, explaining differences between the droplet culture approach and the traditional culture, are given in **Figure 5**, with most of them increasing in abundance in the droplet method. It is worth noting that the single-cell droplet approach recovered largely uncultured *Thiotrichaceae* (i.e., the reference sequence obtained through genomics), illustrating its promise in the discovery of yet-to-be-cultured species.

Because we have taken a shorter amplicon region for 16S rRNA sequencing, to obtain a better taxonomic resolution of the species, we randomly picked 100 colonies from three plates under each condition for Sanger sequencing with universal primers 27F and 1492R (**Tables 1**, **2**). A total of 7 species were found from the traditional culture (**Table 1**), but 14 were found from the droplet culture (**Table 2**). There were 5 species common between the two groups (**Figure S5**). These results are in excellent agreement with the overall 16S rRNA characterisation. Together, they show that our single-cell droplet culture approach provides a facile and effective platform for culturing gut microbiota and preserving its diversity.

**Table 1:** Result of the obtained species using the traditional method.

Isolates ID^a^	Total isolates^b^	Species	Genus	Similarity^c^
1, 2, 5, 18, 34, 47, 51, 63, 90, 91	10	*E. faecalis*	*Enterococcus*	100%
13, 14, 17, 46, 81, 82, 92	7	*E. gallinarum*		99.72%
3, 27	2	*L. reuteri*	*Lactobacillus*	99.31%
7, 10, 12, 15, 19, 60, 68, 85, 86, 87, 93, 94, 95	13	*L. johnsonii*		99.79%
6, 25, 26, 29, 30, 31, 32, 33, 35, 36, 38, 39, 40, 41, 42, 43, 44, 45, 48, 49, 50, 52, 53, 55, 56, 58, 59, 61, 62, 65, 66, 67, 69, 70, 71, 72, 80, 96	38	*P. aquatica*	*Pelomonas*	99.93%
28, 37	2	*P. saccharophila*		99.78%
64	10	*S. roterodami *	*Staphylococcus*	99.72%

a : Isolates ID. – the reference number of each colony.

b : The total isolates obtained that belong to the same species.

c : A similarity is a number used to describe how similar the query sequence is to the target sequence. The higher the similarity, the more significant the matching (Pearson, 2013). The 16S rRNA gene sequences of the isolates in Table 1 have been deposited in GenBank databases under the accession numbers ON974135-ON974208.

**Table 2:** Result of the obtained species with droplet encapsulation.

Isolates ID	Total isolates	Species	Genus	Similarity
5, 8, 24, 29	4	*B. paraconglomeratum*	*Brachybacterium*	100%
36	1	*B. cereus*	*Bacillus*	100%
42, 46	2	*B. licheniformis*		99.12%
17, 41	2	*B. mojavensis*		99.90%
6, 7, 10, 45	4	*B. casei*	*Brevibacterium*	99.93%
44	1	*B. sanguinis*		99.69%
84, 94, 26	3	*E. faecalis*	*Enterococcus*	100%
23	1	*E. gallinarum*		99.65%
70, 71	1	*P. aquatica*	*Pelomonas*	99.93%
52, 85, 88	3	*L. johnsonii *	*Lactobacillus*	99.79%
74, 75, 76, 77, 78, 80, 81, 82, 83, 86, 87, 89, 90, 91, 92, 93, 47, 48, 49, 50, 51, 53, 54, 56, 57, 58, 59, 60, 61, 62, 63, 64, 65, 66, 67, 68	36	*L. murinus*		99.72%
79, 55	1	*L. reuteri*		99.66%
1, 4, 19, 27, 40, 43	6	*M. endophyticus*	*Micrococcus*	99.93%
2, 12, 13, 15, 16, 18, 20, 21, 22, 28, 30, 31, 32, 33, 35, 37	16	*M. luteus*		99.93%

The 16S rRNA gene sequences of the isolates in Table 2 have been deposited in GenBank databases under the accession numbers ON974317-ON974397.

### Isolating gut bacteria metabolically associated with EBPB

With the advantages demonstrated by the single-cell droplet culturing approach, we next exploited this platform to isolate gut bacteria capable of using butyrate or other metabolites produced by the EBPB bacteria (Bai et al., 2020). Oral administration of the EBPB bacteria alleviated obesity symptoms, and the metagenomic study revealed an altered gut microbiota (Wang et al., 2022). To illustrate the mechanism, it is necessary to understand who interacts with EBPB in the gut microbiota.

Here, we spread the collected droplets on the conditioned medium plates (CMP, i.e. containing the EBPB supernatants) under the same seeding condition as that on the YCFA plate Similarly, all colonies (denoted as **E**) were scraped for 16S rRNA amplicon sequencing. Although the alpha diversity of the obtained community **E** appears different from the others, there was no significant difference (**Figure 4A**) from the original communities **C** or **L**. Beta-diversity analysis reveals an independent cluster for community **E**, suggesting their unique composition (**Figure 4B**). Interestingly, *Lactobacillus* is the most abundant genus in the community, and *Bifidobacterium* is within the top 10 (**Figure 4C**). These confirm our previous *in vivo* study, implanted EBPB in mice enriched *Lactobacillus* and *Bifidobacterium* (Bai et al., 2020; Wang et al., 2022).

Similarly, we further characterised randomly picked 100 single-cell colonies for species identification *via* Sanger sequencing. 11 species under four genera (namely, *Lactobacillus*, *Bifidobacterium*, *Bacillus*, *Enterococcus*) were identified (**Table 3**). Among the assigned 86 isolates, 42 belong to *Lactobacillus* (i.e. the top genus), and one belongs to *Bifidobacterium* (i.e. *Bifidobacterium pseudocatenulatum*), which agrees well with the 16S rRNA study. It is worth noting that *Bacillus subtili genus* is the 2^nd^ largest group (33 isolates). It has been shown recently that *Bacillus subtilis* can produce bifidogenic factors that promote the growth of *Bifidobacterium* species (Hatanaka et al., 2020).

**Table 3:** Result of the obtained isolates with droplet encapsulation using the CMPs.

Isolates ID	Total isolates	Species	Genus	Similarity
17,18,21,22,23,36,37,38,49, 56, 57, 70, 71, 75, 76,77, 78, 79, 80, 84, 85, 86, 88, 90, 91, 97	26	*L. murinus*	*Lactobacillus*	99.93%
50, 58	2	*L. intestinalis*		100%
51, 61, 64, 65, 66, 67	6	*L. vaginalis*		100%
53,54, 59, 60	4	*L. reuteri*		100%
48, 55, 62, 63	4	*L. johnsonii*		99.79%
52	1	*B. pseudocatenulatum*	*Bifidobacterium*	99.79%
1, 3, 41	3	*B. cereus*	*Bacillus*	100%
5, 7, 10, 11, 12, 15,16,19,20, ,24,25,26,28,29,30, 31,32,33,35, 39,40,42,72,73,74,81,82	27	*B. nealsonii*		100%
8	1	*B. paramycoides*		100%
44, 83	2	*B. circulans*		100%
27, 34, 46,47,87, 89, 92, 94, 98, 99	10	*E. faecalis*	*Enterococcus*	100%

The 16S rRNA gene sequences of the isolates in Table 3 have been deposited in GenBank databases under the accession numbers ON974744-ON974829.

## Discussions

The human gut is inhabited by diverse microbes that play a fundamental role in maintaining the health of the host (Clemente et al., 2012). An increasing amount of evidence reveals that many diseases often involve significant variations in the diversity and composition of gut microbiota (Marchesi et al., 2016; Liu et al., 2021). Understanding the underlying process requires the knowledge of “who does what in the gut microbiota” and “how they interact with each other and the host” (Miyoshi et al., 2020). However, the complexity of gut microbiota *in vivo* is prohibitively challenging. To date, our insights come mainly from extensive research of faecal samples, which are used as a surrogate for the gut. Although molecule-based approaches can reveal the genetic composition of the gut microbiota, obtaining pure gut bacteria isolates is indispensable for deciphering the role of specific bacteria and their interactions (Bäckhed et al., 2012). However, traditional culture is time-consuming and biased toward dominant, fast-growing bacteria in the community (Watterson et al., 2020). Even the recent development of “culturomics”, which uses multiple culture conditions, has discovered hundreds of new microorganisms (Lagier et al., 2015), substantially amount of bacteria in gut microbiota have yet-to-be-cultured to allow *in vitro* investigations of their physiologic functions.

With the ability to isolate single cells in a confined environment, droplet-based microfluidic has rapidly become a promising, high throughput tool for microbial cell culture. However, the implementation of this technology for anaerobic bacteria studies is restrained by the difficulties of operating bulky instrumentation in an anaerobic workstation (Kaminski et al., 2016; Liu and Walther-Antonio, 2017). To overcome those problems, we have developed an easy-to-operate microfluidic approach for isolating functional bacteria from gut microbiota. The workflow of single-cell encapsulation in droplets and droplet culture on standard plates can be easily carried out in an anaerobic chamber without sophisticated and bulky instrumentation. This approach also simplifies the interface between the microscale world with the conventional macroscopic operation and thus can be readily implemented in microbiology labs. We showed that the single-cell droplet culture promoted cell growth, especially for the slow-growing and challenging anaerobic cells, which resulted in a significantly higher diversity of the obtained community compared with the traditional approach and preserved the diversity of the original gut microbiota.

**Figure 6:**
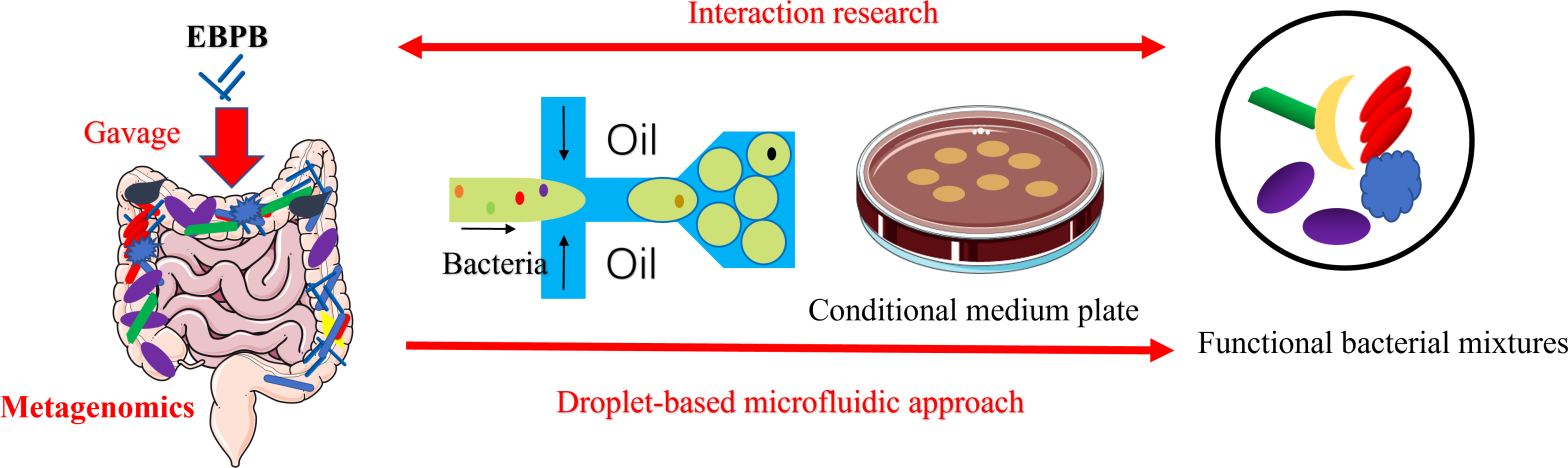
Schematic of the combined metagenomics and the single-cell droplet culture approach to investigate the potential anti-obesity potential of EBPB. The metagenomic analysis of the gut microbiota revealed that the abundance of beneficial bacteria increased after the long-term use of the EBPB. However, isolating, culturing, and analysing the associated bacteria allow us to further study the interaction between EBPB and host, revealing its therapeutic potential. The information will open avenues to develop future living bacteria therapies (e.g., probiotics).

The flexibility of our method for obtaining interactive or metabolically associated bacteria in gut microbiota was illustrated using the butyrate-producing EBPB bacteria, which demonstrated potential in preventing obesity and improving metabolic function (Bai et al., 2020). Our previously metagenomic studies showed that oral administration of EBPB in mice seemed to increase the abundance of beneficial bacteria, such as *Bifidobacterium* and *Lactobacillus, in vivo* (Bai et al., 2020; Wang et al., 2022). Here, the single-cell droplet approach enabled us to obtain the isolates of these beneficial bacteria, providing solid evidence for observed therapeutical benefits. The isolates could be used for further investigations, mining probiotics and constructing artificial flora to develop bacterial therapies against obesity. Taken together, the single-cell droplet culture approach complements culture-independent metagenomic investigations in studying living bacteria therapy. While the metagenomic analysis of the gut microbiota could reveal overall shifts in microbiota composition, isolating gut bacteria and those closely associated are important for further research to understand their function and interaction with the host (**Figure 6**).

## Conclusions

We developed a single-cell droplet on plate culture to isolate and enrich functional gut bacteria from faecal microbiota. The whole process can be easily operated in an anaerobic chamber, allowing the search for obligate anaerobic bacteria. We show the reliable formation of single-cell colonies and significantly improved diversity and evenness of the obtained species. The approach integrates the capability of microfluidics for high throughput and precise cell manipulation with the simplicity of plate culture and can be easily implemented in traditional microbiology labs. With this approach, we have successfully obtained pure gut bacteria isolates that are metabolically associated with the engineered EBPB bacteria, shining a light on the mechanism of its therapeutical potential. We show that our approach, in combination with metagenomic studies, will provide a powerful tool to study gut microbiota and develop potential therapeutics (e.g., probiotics).

## Data availability statement

The original contributions presented in the study are publicly available. This data can be found here: GenBank ON974135-ON974208; ON974317-ON974397; ON974744-ON974829; NCBI - BioProject PRJNA861917

## Author contributions

HH, HY, PW, GK, YS, XL, JY, and XC conceived and designed the experiment; YS fabricated the silicon mould. XC and JY performed the experiments; XC, JY, and UI analyzed the data. JY, XC, HH, and HY wrote the manuscript. XC and JY contributed equally to this work. All authors read and approved the final manuscript.

## Funding

We acknowledge the support from the National Key Research and Development Project (No. 2019YFA0905600), Science and Technology Program of Tianjin, China (No.19YFSLQY00110), Shaoxing “Ming Shi Zhi Xiang” Meritocrat Project and EPSRC IAA (Glasgow). UI is further supported by EPSRC (EP/P029329/1 and EP/V030515/1).

## Acknowledgments

The DNA model elements in **Figure 1** were sourced from Scidraw.io. Furthermore, the part original elements of the **Figures 1**, **6** were drawn by using pictures from Servier Medical Art. Servier Medical Art by Servier is licensed under a Creative Commons Attribution 3.0 Unported License.

## Conflict of interest

Authors XL and PW were employed by Tianjin Modern Innovative TCM Technology Co. Ltd.

The remaining authors declare that the research was conducted without any commercial or financial relationships that could be construed as a potential conflict of interest.

## Publisher’s note

All claims expressed in this article are solely those of the authors and do not necessarily represent those of their affiliated organizations, or those of the publisher, the editors and the reviewers. Any product that may be evaluated in this article, or claim that may be made by its manufacturer, is not guaranteed or endorsed by the publisher.
