# Supplementary material for: A droplet-based microfluidic approach to isolating functional bacteria from gut microbiota
Source: Front Cell Infect Microbiol. 2022 Aug 18;12:920986. doi: 10.3389/fcimb.2022.920986 (PMC9433703; doi:10.3389/fcimb.2022.920986)
Supplement: Supplementary file 1 [file DataSheet_1.docx]

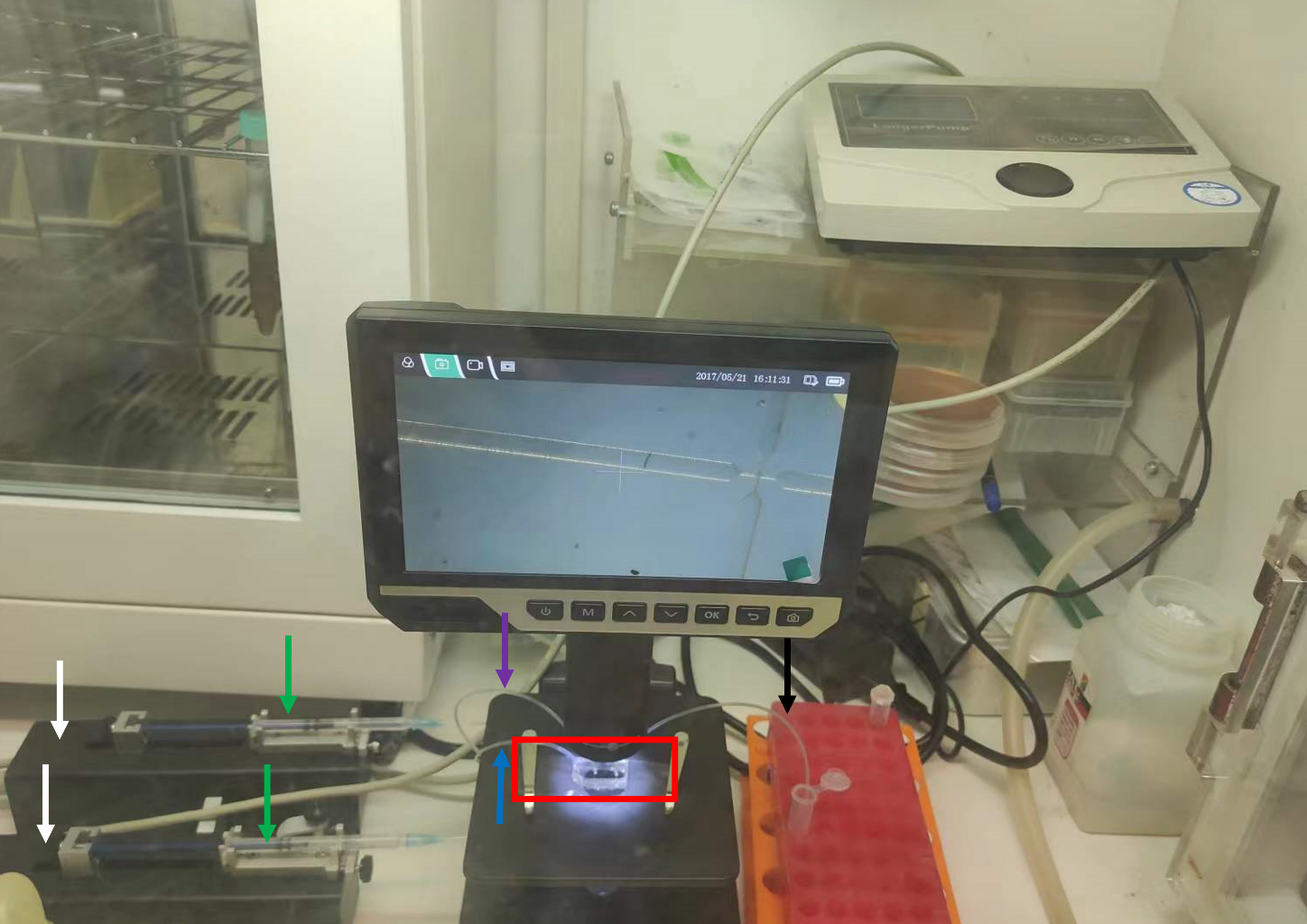


**Figure S1: The setting for encapsulating single bacteria cells in droplets.** Two pumps (white arrows) control the flow rates from two syringes (green arrows) containing either bacteria or mineral oil. Tubing delivers bacteria (purple arrow) and oil (blue arrow) from the syringes to the microfluidic chip (red rectangle). The third piece of tubing delivers the formed droplets to a collection tube (black arrow). This system is placed in an anaerobic chamber.


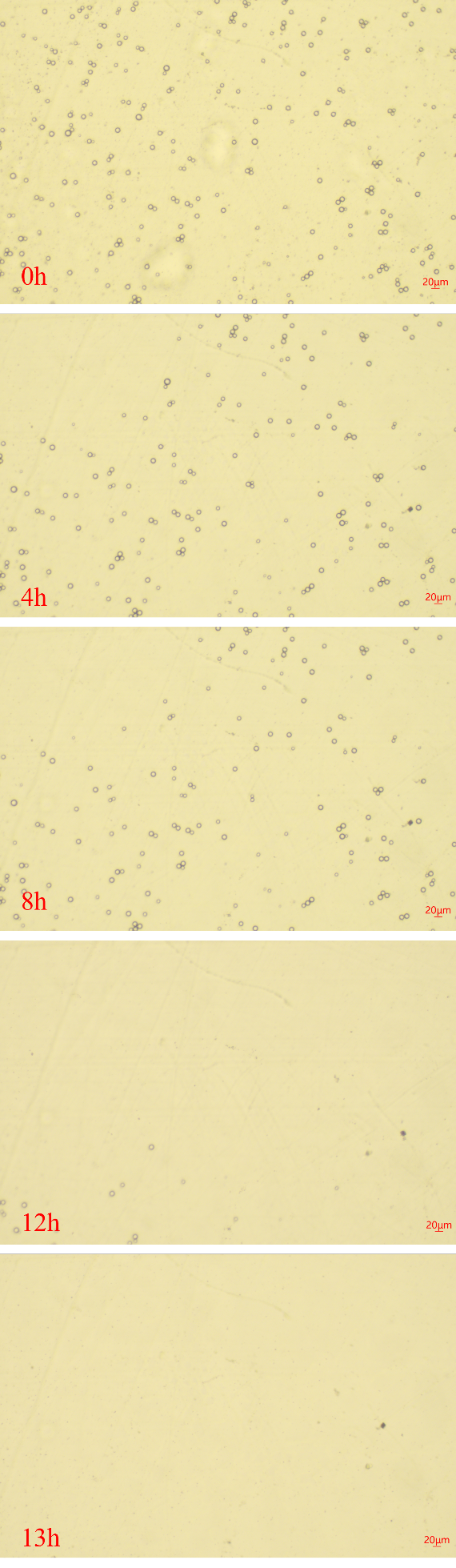


**Figure S2: Time-lapse images of droplets on an agar plate at 37°C. Scale bars, 20 μm.**


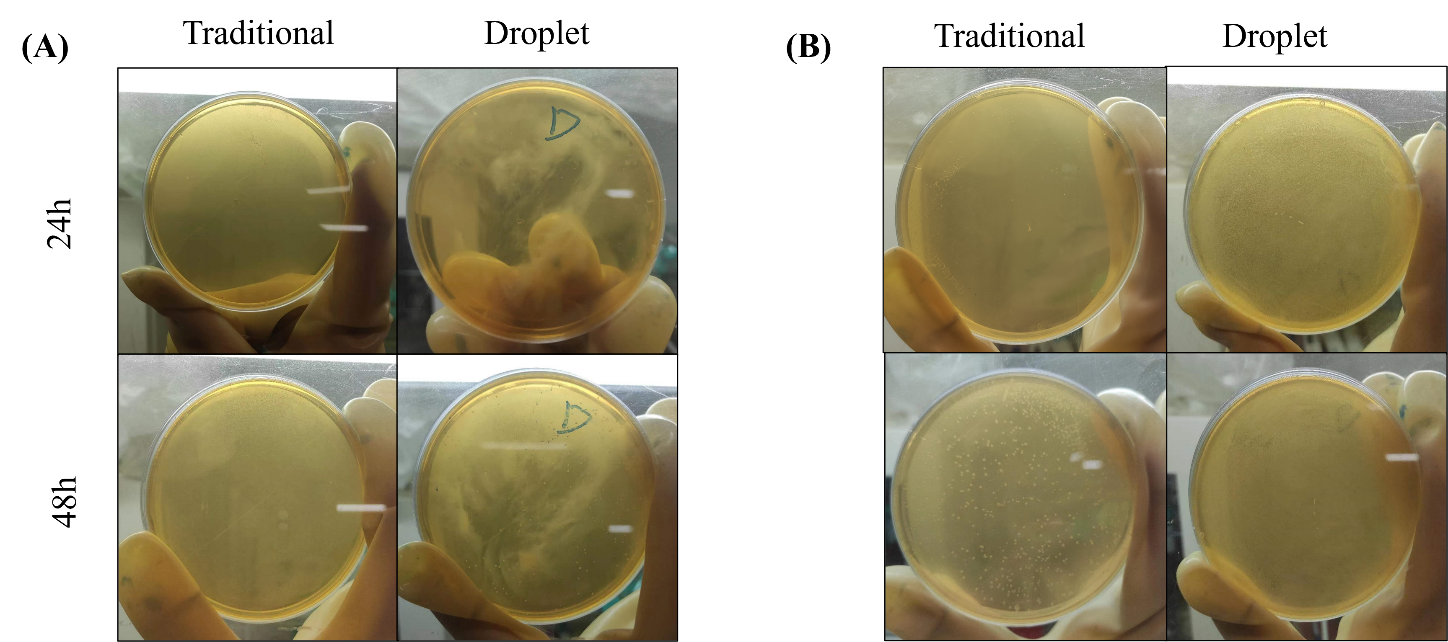


**Figure S3: Culturing of** ***E. coli* and *B. pseudocatenulatum* with or without single-cell droplet encapsulation.** (A) *B. pseudocatenulatum* with droplet encapsulation grew on agar plates after 48h culture but did not grow on plates using the traditional plate method. (B) *E. coli* grew on agar plates either with or without droplet encapsulation after 48h culture.

**
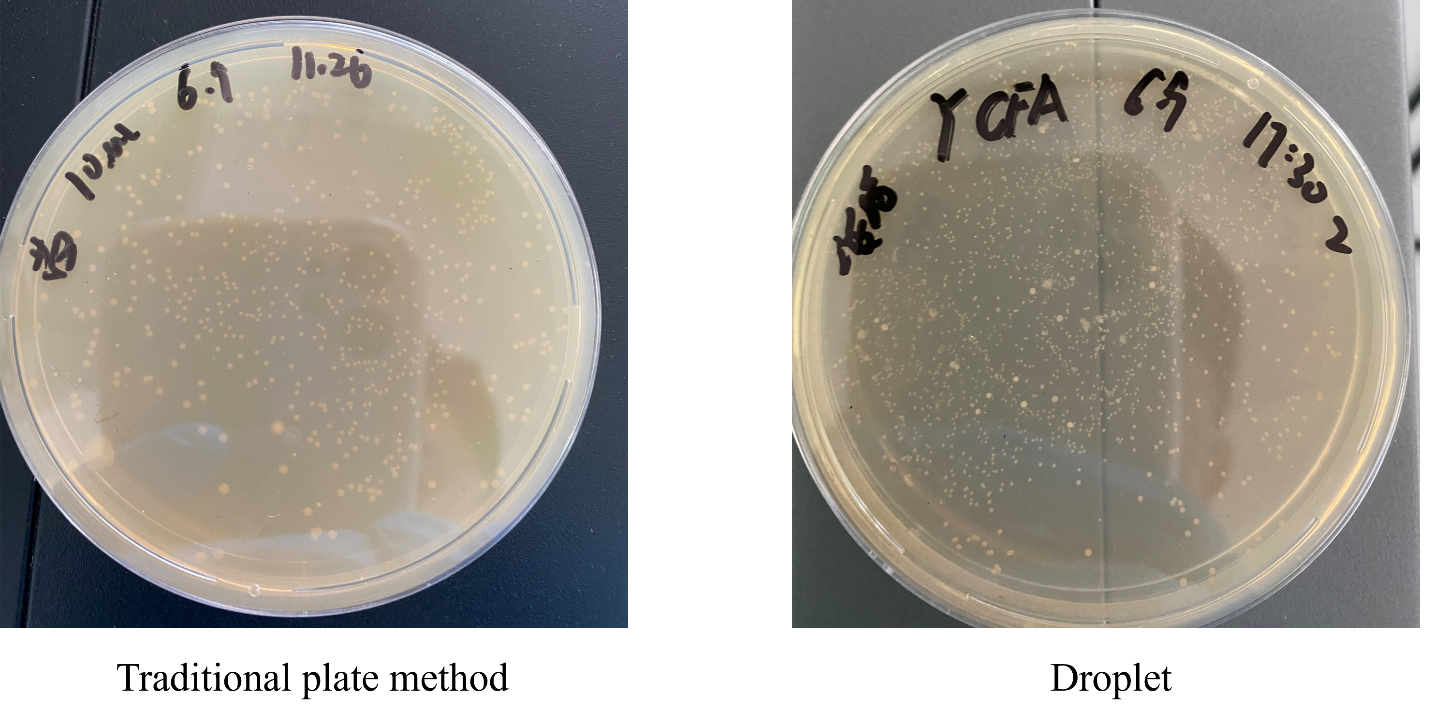
**

**Figure S4:** Colony formation numbers for traditional and droplet methods after 72 hours of culture.


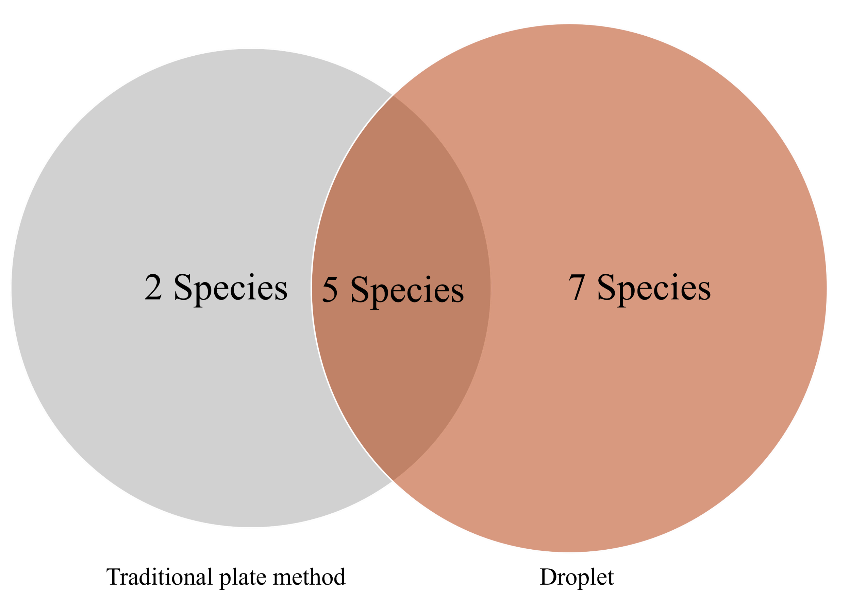


**Figure S5. Venn diagram of species by picking a single colony and then Sanger sequencing.**
